# Morphological studies of labyrinthine tissue in patients affected with Meniere’s disease and vestibular schwannoma following labyrinthectomy

**DOI:** 10.1007/s00405-024-09160-4

**Published:** 2024-12-26

**Authors:** Ankit Ajmera, Nikeith John, Adrienne Morey, Nigel Biggs, Sean Flanagan, Peter Earls, Daniel Brown, Payal Mukherjee

**Affiliations:** 1 ENT Department, Sydney Adventist Hospital, Sydney, NSW Australia; 2 Department of Anatomical Pathology, ACT Pathology, Canberra Hospital, Canberra, ACT Australia; 3 ENT Department, St. Vincent’s Hospital, Sydney, NSW Australia; 4 SydPath Anatomical Pathology, St Vincent’s Hospital, Sydney, NSW Australia; 5 Curtin University, Perth, WA Australia; 6 The University of Sydney, Sydney, Australia

**Keywords:** Meniere's disease, Vestibular schwannoma, Immunohistopathology, Pathology

## Abstract

**Background:**

Meniere’s disease (MD) is a disabling disease of the inner ear, having a substantial effect on a patient’s quality of life. While various postulations regarding its aetiology exists, due to the difficulty with accessing inner ear tissue, there have been limited histological studies in patients with active MD.

**Methods:**

Tissue was collected during labyrinthectomy from 8 patients with intractable MD who had failed medical therapy (22 samples), and 9 patients undergoing translabyrinthine resection of vestibular schwannoma (19 samples). 20 additional samples were obtained from 2 cadavers without a history of inner ear disease. Samples were assessed with routine histology and a panel of immunohistochemical markers to assess any differences between the groups.

**Results:**

No MD samples demonstrated significant inflammatory infiltrate, evidence of denervation of the sensory epithelium, fibrosis, or thickening of blood vessel wall stroma. Novel findings included confirmation that no lymphatic channels of usual type were present and that the subepithelial stromal cells are strongly positive for S100, suggesting possible perineurial origin. There were no consistent differences in expression of Claudin or Aquaporin between the MD and VS patient samples.

**Conclusion:**

This is one of the largest comparative histological study utilising operative samples from inner ear of living donors with active intractable MD and control patients with VS. There were no significant morphological differences between the two groups, suggesting that the aetiology lies elsewhere within the vestibular system. Examination of endolymphatic sac tissue is therefore a priority for future work.

## Introduction

Meniere’s disease (MD) is a chronic destructive and disabling disease of the inner ear, defined by symptoms including (a) the presence of 2 or more attacks of vertigo lasting 20 min to 12 h (b) low frequency hearing loss, (c) fluctuating aural fullness (d) tinnitus and (e) no other obvious vestibular cause [1]. Attacks occur episodically with periods of normal inter-episodic functioning, however, the cumulative impact of the disease results in varying degrees of cochlear and vestibular damage [2]. The unpredictability, combined with the audiovestibular disability, can have substantial effect on the quality of life impact of those affected [3]. 

There are several theories regarding the underlying aetiology of MD, however, a consistent hallmark in MD is endolymphatic hydrops [4]. This may be due to homeostatic dysregulation of fluid volume within the inner ear, either through the overproduction or the under-resorption of endolymph fluid [4]. Other postulations implicate an inflammatory or autoimmune component, as well as abnormal expression of proteins involved in fluid regulation as the underlying cause [5]. Caloric and head impulse testing in MD have also been shown to be discordant [6] with suggestions that an increased diameter in the horizontal semicircular canal may result in an altered endolymph circulation [7, 8]. 

While a limited number of prior histological studies have tried to identify the morphological abnormalities in MD, the challenges in accessing labyrinthine tissue for histological analysis have led to a paucity of reports correlating physiological changes observed in vestibular testing with histology of the patient’s labyrinthine tissue. Furthermore, many of these studies either had no controls, or utilised cadaveric samples from MD patients, the latter potentially affected by tissue autolysis and decalcification artifact. When samples are harvested from surgical procedures, potential crush artifact, as well as disorientation of the membranous labyrinth adds additional complexity to tissue interpretation.

The primary objective of the present study was to compare the morphological features of the vestibular membranous labyrinth in surgical samples from patients with active MD with control samples from patients undergoing labyrinthectomy for vestibular schwannoma (VS), including immunohistochemical analysis for a subset of proteins involved in fluid regulation. Implications of the findings for understanding the underlying pathogenesis of MD were considered. As there is currently limited conclusive histological evidence regarding the morphology and pathophysiology of MD, the study attempts to overcome issues that exist with previous studies by utilising a larger sample of human tissue for sampling.

## Methods

Human Research and Ethics Committee approval was obtained from Research Ethics and Governance Information System, NSW Health protocol number 2019/ETH09627 for prospective tissue collection during labyrinthectomy of patients with intractable MD and for those undergoing translabyrinthine approach for VS resection as the control arm. Patients in the MD group met the amended 2015 Criteria for the diagnosis of MD.

### Sample selection

A total of 61 separate histological samples were obtained from 17 patients (adults over the age of 18) who were prospectively recruited over a 3-year period undergoing labyrinthectomy for intractable MD (8 patients; 22 samples) or curative surgery for VS (9 patients; 19 samples). 6 of the submitted samples were deemed to have inadequate material for histological assessment. Additionally, 2 (previously frozen) cadaveric temporal bones without a history of inner ear disease provided 20 additional samples. These samples provided a control for surgical sampling error and were retrieved in a controlled environment to minimise surgical crush artefact. See Table 1 for breakdown of samples collected.

**Table 1:** Summary of tissues obtained for histological analysis

	Meniere’s disease	Vestibular schwannoma	Cadaveric temporal bone	Total
Total patients	8	9	2	19
Lateral semicircular canal	3	6	4	13
Posterior semicircular canal	5	1	4	10
Superior semicircular canal	5	4	4	13
Non-specified canal	0	2	4	6
Vestibule/Utricle	9	6	2	17
Endolymphatic sac	0	0	2	2
Total samples	22	19	20	61

### **Fixation and** routine staining

All tissue samples were fixed in neutral buffered formalin and embedded in paraffin. This was chosen as it affords flexibility with permanent retention of tissue and availability for immunohistochemical analysis with antigen retrieval as required. No decalcification processing was employed.

Routine haematoxylin and eosin staining was utilised to demonstrate morphology of the different components of the inner ear, and to assess for any differences in basement membrane thickness, hair cell density, inflammatory cell infiltrate and dystrophic calcification between the MD and VS patients.

### Immunohistochemistry

A sub-group of 7 patients with better preserved detailed samples were chosen for more detailed immunohistochemical analysis (3 MD patients and 4 VS Patients). Samples were assessed using a panel of routinely employed antibodies against various structural elements and cell-type specific antigens (pan-keratin [AE1/AE3], S100, D2-40, ERG, calretinin, glial fibrillary acidic protein [GFPA], neurofilament and CD35), as well as non-routine antibodies against aquaporins 1, 4 and 6 (water transporter molecules) and claudins 3 and 4 (tight junction proteins) (Table 2) due to their role in fluid regulation. The latter non-routine antibodies required validation with control tissues and protocol optimisation before application to study materials. Appropriate positive control sections were cut onto all slides prior to staining. Immunostaining was performed on Ventana Ultra platform with CC1 antigen retrieval (AR). Immunohistochemistry was not undertaken on the cadaveric samples because of the marked autolytic changes evident on routine histology.

**Table 2:** Antibodies to fluid-handling proteins

ANTIBODIES	Supplier / protocol	Role
Aquaporin 1	Abcam ab9566; 1/100 with CC1 AR	Forms water-specific channel that provides the plasma membranes of a variety of cells (including red cells and kidney proximal tubules) with high permeability to water
Aquaporin 4	Abcam ab9512; 1/100 with CC1 AR	Forms water-specific channel and acts as osmoreceptor regulating body water balance and mediating water flow within the CNS
Aquaporin 6	Abcam ab191061; 1/100 with CC1 AR	Forms a water specific channel that participates in distinct physiological functions such as glomerular filtration, tubular endocytosis and acid-base metabolism and is localised in cytoplasmic vesicle membranes
Claudin 3 [ERP19971]	Abcam ab214487; 1/250 with CC1 AR	Located on the cell membrane and plays a major role in tight junction-specific obliteration of the intercellular space through calcium independent cell-adhesion activity
Claudin 4 [ERP17575]	Abcam210796; 1/4000 with CC1 AR	Located on the cell membrane and plays a major role in tight junction-specific obliteration of the intercellular space through calcium independent cell-adhesion activity

## Results

### Routine histology

**Fig. 1:**
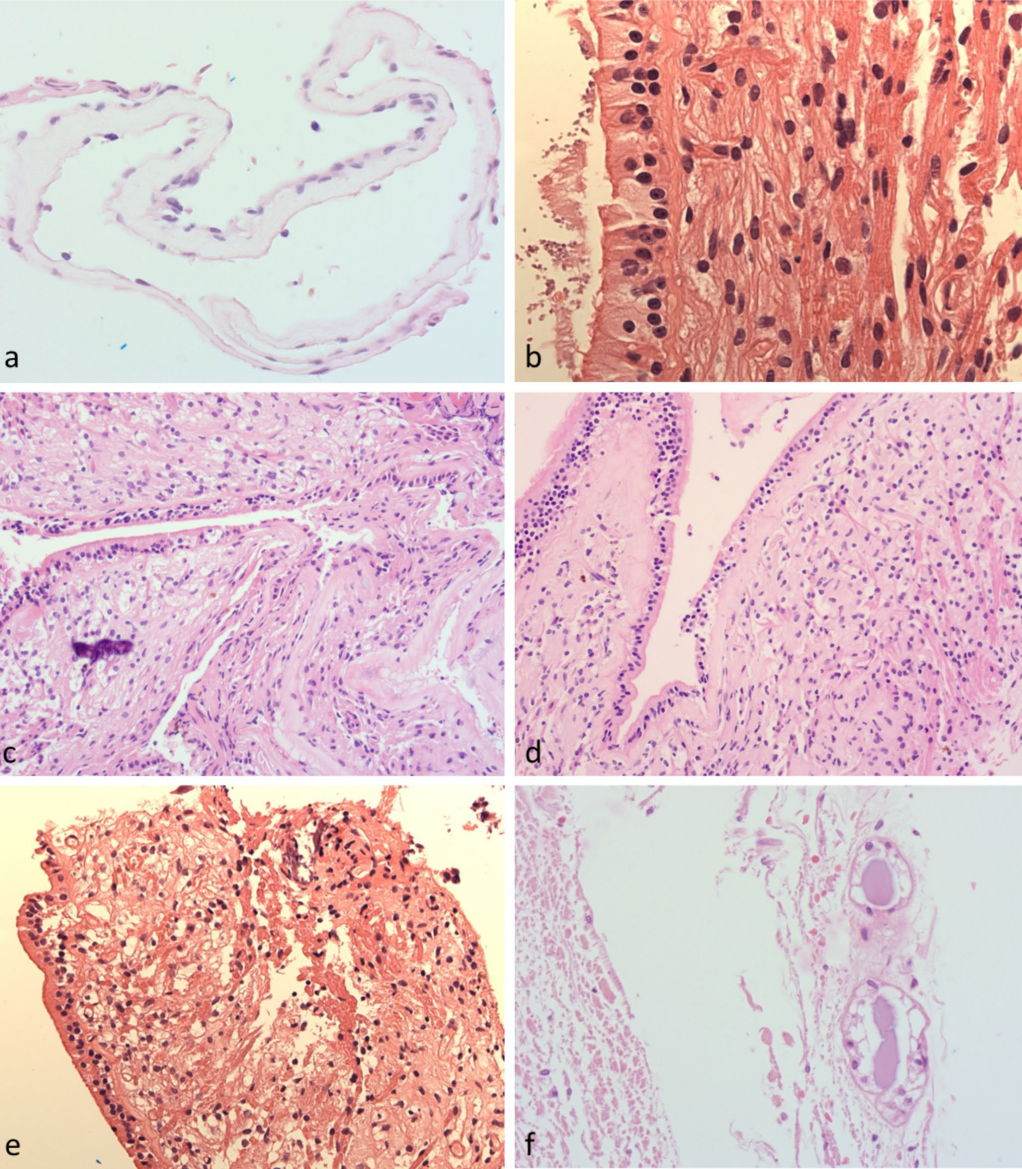
Histology: (**a**) Peripheral canal samples showing attenuated cells over thick basement membrane (**b**) Sample from lateral semicircular canal of VS patient with thin basement membrane (**c**) Sensory epithelium showing increasing thickness of basement membrane (**d**) Post gentamicin patient (**e**) Nerve fibres traversing stroma in VS (**f**) Endolymphatic sac from Cadaveric sample

Many samples from the semicircular canals consisted only of collapsed tubular structures lined by attenuated epithelium with a few stripped stromal cells on the other side of a thickened eosinophilic basement membrane (Fig. 1a). The apparent thickness of this basement membrane varied considerably with the degree of “tilt” of the plane of embedding. None of the samples showed ciliation of the lining epithelium of the peripheral canals. The canals are usually attached to the bony labyrinth by thin threads of stroma, which do not survive surgical removal or processing, and are focally bound to the surrounding bone beneath the regions of sensory epithelium.

The largest number of samples with well-preserved sensory epithelium came from the lateral semicircular canals (Fig. 1b) and vestibule. In these samples, a transition from sensory columnar epithelium to adjacent attenuated “tubal type” epithelium was present. The thickness of the basement membrane beneath the attenuated peripheral epithelium was greater than that beneath the sensory epithelium (Fig. 1c) in both MD and VS groups, with a transition zone of variable thickness at the edge of the regions of sensory epithelium. In only one of the MD patients (previously treated with gentamicin) there was thickening of the basement membrane below the lateral canal sensory epithelium (Fig. 1d). The distribution of different cell types also varied considerably between the edge of the sensory epithelium and the more central regions. None of the samples showed overt degenerative or inflammatory changes in the epithelium.

The appearance of the stroma beneath the sensory epithelium was similar in the canal cristae and the maculae, and identical between the MD and VS groups. The appearance of this spongy subepithelial stroma is histologically unique, with tiny, thin-walled inconspicuous vessels and stromal cells of uncertain histiogenesis. No thick-walled vessels were seen in any of the samples, and no vessel wall inflammatory infiltrates or deposits were seen. Tiny vessels in MD patients were no more ectatic than in controls and none of the samples showed any significant inflammatory infiltrate within the stroma or hyaline eosinophilic deposits in the sub-epithelial stroma (which, suggests no abnormal cochlin deposition). The degree of sub-epithelial lipofuscin-like pigment varied between samples but was not more noticeable in the MD samples.

Nerve fibres traversing the stroma (Fig. 1e) generally appeared to be normal, although in four VS samples there was focal dystrophic calcification. Further, one MD sample had focal dystrophic calcification in the stroma beneath the posterior canal sensory epithelium and a second MD sample showed dystrophic calcification immediately adjacent to the basement membrane of a peripheral canal sample, which may possibly be a non-specific age-related change.

Analysis of the samples from cadaveric samples showed similar overall morphology but extensive autolysis and stripping of the epithelium. Ectasia of small vessels in the superficial stroma was more pronounced than in the operative specimens. No inflammatory changes were seen. Considerable lipofuscin-like pigment was deposited immediately beneath the epithelium were seen in multiple samples. Samples from the cadaveric endolymphatic sacs showed scant tubules with autolytic cuboidal epithelium immediately adjacent to the dura (Fig. 1f). Many of the tubules contained eosinophilic proteinaceous secretions without evidence of inflammation.

### Immunohistochemistry

**Fig. 2:**
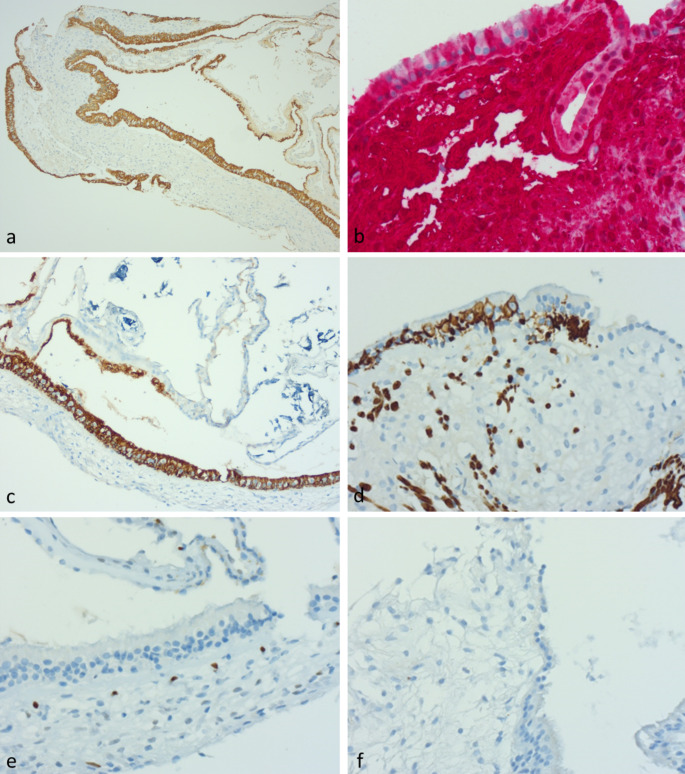
Immunohistochemical staining of VS lateral canal sensory epithelium: (**a**) Pan-keratin (**b**) S100 (red substrate) (**c**) GFAP (**d**) Neurofilament (**e**) ERG (**f**) D2-40

A subset of 7 patients (3 MD, 4 VS) with better preserved and oriented tissues were selected for more detailed immunohistochemical analysis, using a range of markers to examine epithelial characteristics, the presence of inflammation, the integrity of innervation and the characteristics of vessels in the sub-epithelial stroma. The same samples were then investigated for aquaporin and claudin expression to assess fluid handling characteristics of the epithelium and stroma. There was no consistent difference identified in the immunohistochemical analysis between both groups (Table 3; Fig. 2).

**Table 3:** Results of immunohistochemical staining

Stain	Results
Pan-cytokeratin	Positively labelled in sensory epithelium and attenuated lining epithelium (Fig. 2a)
S100	With exception of round type 1 cells, supporting cells, stromal cells, nerves and peripheral epithelial lining all stain positive suggesting neural origin of vestibular stromal tissue (Fig. 2b)
GFAP	Strong staining in supporting cells (except type 1 cells). This diminished as the epithelium became simpler at the edge of the innervated epithelium, then disappeared in peripheral attenuated epithelium (Fig. 2c)
Neurofilament antibody	The nerve fibres stained traversed the spongey stroma and entered the epithelium between the epithelial cells. They surrounded type I cells (forming basket-like calyces) in the central part of the sensory epithelium, but the number of such calyces reduced at the periphery (Fig. 2d)
Calretinin	Did not stain any epithelium or stromal cells but a few specks were seen within nerve fibres
ERG	A network of microscopic vessels not evident on routine stains was arrayed close beneath the basement membrane with no vessel wall thickening or abnormality of the subepithelial vascular meshwork. ERG staining in stromal cells was weak, similar to the degree seen in lymphoid cells. No staining was seen in other fibroblastic cells. (Fig. 2e)
D2-40	Negative in all specimens (Fig. 2f), indicating no “typical” lymphatic channels were present in the stroma of the vestibular system
CD 35	Negative in all specimens
Aquaporin 1	Weak non-specific staining of superficial sensory epithelium in all samples. There was no staining in the stroma (Fig. 4a).
Aquaporin 4	Appeared to show only non-specific background staining in the apical portions of sensory epithelial cells
Aquaporin 6	Moderately strong apical granular cytoplasmic staining in the supporting cells of the sensory epithelium (but not the type I cells) (Fig. 4b). The staining in the sensory epithelial cells was stronger than in the adjacent attenuated tubal lining epithelium. Note, one MD patient (with previous gentamicin treatment) showed a lesser degree of staining, but it is unclear what region was assessed.
Claudin 3	Sensory epithelium was essentially negative (Fig. 4c). Only a weak hint of staining was seen in the apical portion of cells at the edge of the sensory epithelium.
Claudin 4	Moderately strong expression was seen in attenuated canal epithelium of all patients (Fig. 4d), particularly at the edges of sensory epithelium. The sensory epithelium itself generally showed weak staining, the exception being one patient with MD (post gentamicin), where stronger membranous staining was seen in supporting cells in posterior canal epithelium

## Discussion

The central postulation of hydrops in MD suggests a link between fluid homeostasis of the vestibular system and the clinical manifestations of vertigo and aural fullness [7]. While the cause of this abnormal fluid homeostasis is unknown, it has been variously postulated to be linked to abnormal expression of aquaporins or claudins, degradation of ‘adhesive’ vascular endothelial cells, thickening of the basement membrane or abnormal deposits (such as cochlin) within the supporting stroma [5]. This study utilised specimens collected from surgical patients, to compare the histology of the membranous labyrinth between MD and VS (control) patients. It is one of the largest comparative studies of MD histology using non-cadaveric material and builds on the evidence behind the aetiology of MD.

### Histology

Our morphological investigations have not demonstrated any unique abnormality in the sensory epithelium or underlying stroma of patients undergoing surgery for intractable MD. There was no evidence of acute or chronic inflammation, fibrosis or denervation. No abnormal deposits were seen within the stroma. Perhaps these findings are not unexpected, as for patients to experience acute symptoms, they need to have an intact sensory apparatus and be able to transmit neural signals. In this study, all MD tissue was collected during labyrinthectomy in the setting of unremitting symptoms of MD. Intact receptor morphology supports the theory that the epithelium is suffering episodic overstimulation due to some mechanical event within the labyrinth, such as a sudden fluid shift between compartments, rather than intrinsic damage to the neuroepithelium.

### Basement membrane and neuroepithelia

McCall (2009) demonstrated non-specific basement membrane thickening, organelle changes, and an absence of hair cells when assessing post-mortem temporal bones [9], which was in keeping with the postulation of cellular stress. It is unclear whether this basement membrane thickening extended into the semicircular canals which are postulated to be involved in the ionic regulation of the endolymph [10, 11] or if this thickening was exclusive to the sensory epithelium. Calzada (2012) further demonstrated a disorganised supportive matrix with neuroepithelial degeneration and a thickened basement membrane in MD temporal bones when compared with control specimens with normal audiovestibular functions [12]. 

On the contrary, our study did not consistently find basement membrane thickening and neuroepithelial monolayering in MD specimens unlike McCall and Calzada [9, 12]. We found thickening of the basement membrane below the lateral canal sensory epithelium in only a single MD patient who had been previously treated with gentamicin. Our finding of considerable variation in the thickness of the sub-epithelial basement membrane according to the site (as well as orientation) of the sample (in both MD and VS patients) indicates that care should be taken when interpreting isolated measurements of basement membranes thickness. Additionally, orientation of these tiny fragile samples is difficult, and “tilt” due to plane of embedding may also affect measurement of membrane thickness.

Tsuji identified a selective loss of Type II hair cells and Scarpa’s ganglion cells in the neuroepithelium of MD samples [13]. This was later challenged by McCall [9] and Calzada [12] who noted no preferential loss of Type II vs. Type I hair cells, but rather a vacuolisation of hair cells and supporting cells with a disorganisation of the basement membrane in MD samples. We identified considerable variation in the cellular constituents and staining profile of the sensory epithelium depending on whether the sample was from the centre or the edge of the sensory apparatus, suggesting similar caution must be applied when assessing relative numbers of certain cell types in small (particularly ultrastructural) samples, as the exact location influences epithelial cell composition and innervation.

### Immunohistochemical evaluation of structural elements

Evaluation of the immunohistochemical profile of various structures within the vestibular system identified several novel findings;

i) No lymphatic vessel of “usual” type (i.e. lined by endothelial cells staining for D2-40) were identified within the stroma beneath the vestibular epithelium. This is similar to the central nervous system where there is an absence of true lymphatic vessels, and has implications for fluid handling within the vestibular system.
ii) The subepithelial plump stromal cells showed uniformly strong positivity for S100. This is not a characteristic of lamina propria/fibroblast-containing stroma of other organs and raises the possibility that the stroma is of perineurial origin.
iii) The entire vestibular epithelium stained positively for pan-keratin and (except for type 1 cells) was also strongly positive for S100; supporting cells also labelled for GFAP. The presence of strong epithelial staining for S100 is unusual as it is absent in most normal epithelia.

### Immune/inflammatory features

The absence of significant inflammatory cell infiltrate within the stroma is similar to that reported by McCall [9]. We also demonstrated no expression of CD35 (which labels dendritic cells involved in antigen presentation), no perivascular inflammation, no vessel wall thickening and no eosinophilic deposits in the subepithelial stroma suggesting that local FDC-mediated secretion of cochlin is not involved in the pathogenesis of MD. This is in contrary to Calzada who demonstrated increased cochlin immunoreactivity in the vestibular stroma of patients with MD [12]. The interest in cochlin deposition was based on the work of Robertson (2001, 2006) who demonstrated increased eosinophilic deposits of cochlin in the ampullary stroma of patients with hereditary DFNA9-associated hearing loss and vestibular dysfunction [14, 15]. Furthermore, Py (2013) demonstrated that cochlin plays a role in innate immunity and is secreted by follicular dendritic cells [16].

### Fluid handling

Monsanto (2017) proposed an anatomical predisposition to hydrops and symptom development due to a decreased endolymphatic volume following volumetric analysis of 16 MD, 16 endolymphatic hydrops and 16 non-diseased cadaveric temporal bones [17]. The 3D reconstructions of the vestibular aqueduct and the endolymphatic sac within the temporal bones demonstrated lower volumes in the MD group with a comparatively smaller vestibular aqueduct, suggestive of an anatomical predisposition to developing MD and the associated symptoms.

Ishiyama (2017, 2018) demonstrated tight junction integrity with stromal and perivascular basement membrane disorganisation, oedema of the pericytes and swelling of the blood-labyrinthine barrier (BLB) in vestibular end organs of MD patients [18, 19]. It is unclear if these changes were the cause or effect of the ionic and volume disturbances leading to endolymphatic hydrops (and the resulting symptomatology). Dixon Johns (2023) demonstrated a reduction of KCNJ10 expression, which plays a role in cellular membrane potential, in temporal bone specimens of those with MD [20]. Lopez (2007) demonstrated the presence of aquaporin (AQP) 1, AQP 4 and AQP 6 by immunofluorescence of inner ear samples from non-diseased temporal bones [21]. AQP 1 was noted to be distributed to fibrocytes and blood vessels of the underlying stroma within the cochlear, AQP 4 was localised to the basal pole of vestibular supporting cells in the macula utricle and cristae ampullaris, and AQP 6 was localised to the apical portion of the vestibular supporting cells, the hair cells themselves being non-immunoreactive.

By contrast, in our study staining for AQP 1 did not show specific labelling in any of the vestibular sections, AQP 4 demonstrated non-specific background staining in apical portions of the sensory epithelia and AQP 6 demonstrated strong apical granular cytoplasmic staining in supporting cells of sensory epithelia, but not in adjacent attenuated lining epithelium. No obvious difference was present between MD and VS samples. While the apical localization of AQP 6 was similar to that reported by Lopez [21], the localization of AQP 1 and AQP 4 appears discordant, suggesting that either post-mortem autolysis affected the staining in the cadaveric samples [20], or that antibodies need further optimization for use in paraffin sections.

**Fig. 3:**
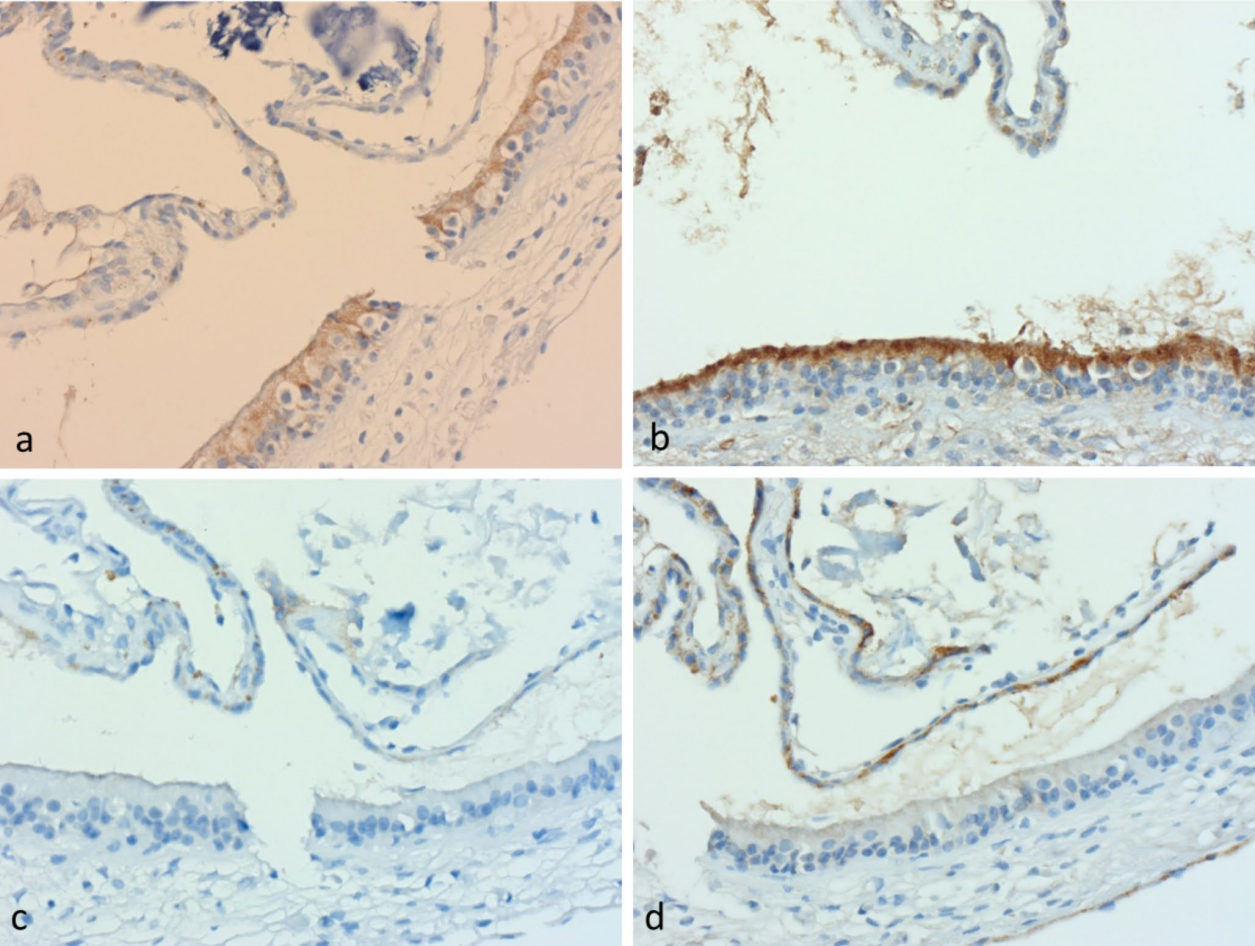
Fluid-handling antibodies of lateral canal of VS (**a**) Antibody to Aquaporin 1; (**b**) Antibody to Aquaporin 6; (**c**) Antibody to Claudin 3 was negative; (**d**) Antibody to Claudin 4

We identified no staining for Claudin 3 in our examinations of both MD and VS samples. Moderately strong staining for Claudin 4 was seen in attenuated canal epithelium, with the sensory epithelium staining weakly in most samples, indicating the presence of preserved tight junctions in both the MD and VS samples. We noted the apparent increased epithelial membranous staining for Claudin 4 in the MD patient with past history of gentamicin therapy; the reason for this is unclear, but it would not appear to support a thesis of “leaky” epithelium. (Fig. 3).

### Study limitations

A limitation of the study was the inability to examine tissues from endolymphatic sac in patients undergoing either labyrinthectomy or vestibular schwannoma resection. This has been a limitation of all reported studies in non-cadaveric samples, as it is not a routine part of transmastoid labyrinthectomy due to the risk of CSF leak. Further analysis of endolymphatic sac tissue in both MD patients and controls would therefore appear to be a priority for future work, to better understand the blood-labyrinth barrier in the sac and its role in MD.

## Conclusion

While a consensus on the underlying pathophysiology of MD has not been reached, our study has provided context to the histopathological changes observed in active MD through its comparison with a control VS cohort using non-cadaveric specimens. Current cadaveric data are generally affected by degenerative and age-related changes, preservation artefact and changes associated with decalcification, which may not represent the features of active disease and thus affect morphological interpretation. Our study avoids this and further demonstrated the importance of tissue preservation to reduce artifact. Further, it identified some unique histological features, confirmed and challenged other findings. While it did not identify any clear differences between MD and VS samples, given the current paucity of non-cadaveric analysis in literature, the study builds on the current evidence by suggesting that the aetiology of abnormal fluid handling may lie elsewhere within the vestibular system. Examination of tissues from the endolymphatic sac from patients with active MD would therefore appear to be a priority for future work.
